# Prospect in Alzheimer’s disease integrative therapy targeting both amyloid-beta and tau

**DOI:** 10.4103/NRR.NRR-D-24-00916

**Published:** 2025-12-30

**Authors:** Wei Zhang, Yiqun Zhou, Jiuyan Chen, Miranda Perez, Xiomara Claure, Roger M. Leblanc

**Affiliations:** 1 Department of Chemistry, University of Miami, Coral Gables, FL, USA; 2 C-Dots LLC, Miami, FL, USA

**Keywords:** Alzheimer’s disease, amyloid-β, integrative therapy, interplay, multi-target therapy, tau

## Abstract

Alzheimer’s disease is well characterized by the buildup of amyloid-β plaques and tau protein tangles, leading to neurodegeneration and cognitive impairments. Recent prosperous Alzheimer’s disease therapeutic development targeting amyloid-β validates the amyloid hypothesis. Nonetheless, the limited efficacy of single-target therapies plus as-observed synergy between amyloid-β and tau calls for a thorough understanding of Alzheimer’s disease pathogenesis. Thus, this review introduces Alzheimer’s disease pathogenesis, specifically focusing on the amyloid-β and tau pathologies, highlights their interconnected nature, presents personal perspectives on therapeutic and diagnostic challenges, and underscores the necessity of combined therapeutic approaches to effectively address Alzheimer’s disease.

## Introduction

**Figure 1:**
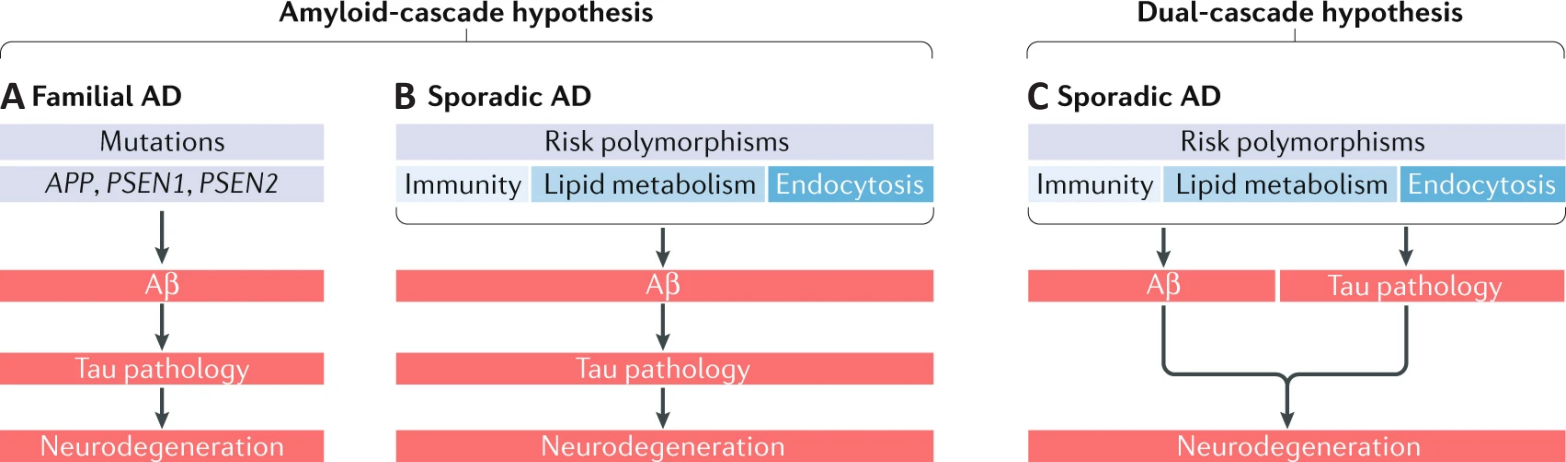
Amyloid hypothesis and dual-cascade hypothesis. (A) Genetic mutations in *APP*, *PSEN1* and *PSEN2* disrupt the amyloidogenic processing of APP and result in familial AD. (B) The amyloid-cascade hypothesis of sporadic AD posits risk factors including polymorphisms in genes but lacks mutations in *APP*, *PSEN1* or *PSEN2*. (C) The dual-cascade hypothesis of sporadic AD suggests that polymorphisms influence Aβ and tau pathologies through interconnected yet distinct cellular pathways. Reprinted with permission from van der Kant et al. (2020). Aβ: Amyloid-β; AD: Alzheimer’s disease; APP: amyloid precursor protein; PSEN1: encoding presenilin 1; PSEN2: encoding presenilin 2.

With the rise of the aging population, by 2030, a notable surge (> 80 million) is expected in the global prevalence of cognitive decline, with Alzheimer’s disease (AD) as the most common type (**~**70%) of dementia (Lozano et al., 2020). AD and its related illnesses have substantial impacts on the patients, caregivers, healthcare systems, and society, which urges the rapid development of effective therapies (2023) and a good understanding of the pathogenesis. The mainstream AD pathogeneses include declined production of acetylcholine, aggregation of amyloid-β (Aβ) and hyperphosphorylated microtubule-associated protein tau, accumulation of reactive oxygen species, neuroinflammation, and genetics (Zhou et al., 2022). As two hallmark features in AD, the aggregations of Aβ peptides and hyperphosphorylated tau into dense fibrillary plaques and neurofibrillary tangles (NFT), respectively, result in neurotoxicity, dendritic shrinkage, synaptic destabilization, and eventual neuronal death (Schneider, 2017). In a recent “ATN” framework, AD is defined based on the fundamental molecular pathology that involves amyloid plaques (‘A’), hyperphosphorylated tau (‘T’) in NFTs, and subsequent neurodegeneration (‘N’). The “ATN” framework asserts that Aβ serves as the primary molecular initiator of tau misfolding, assembly, and spreading, ultimately resulting in neurodegeneration and cognitive decline (**Figure 1A**; Jack et al., 2018).

In the amyloid hypothesis for sporadic AD, while sporadic AD lacks mutations in genes such as *APP* (encoding amyloid precursor protein [APP]), *PSEN1* (encoding presenilin 1 [PSEN1]), and *PSEN2* (encoding presenilin 2 [PSEN2]), both familial and sporadic AD exhibit similar pathological changes in Aβ and tau (**Figure 1B**; De Strooper and Karran, 2016). The amyloid hypothesis for both familial and sporadic AD proposes that the initial abnormality in Aβ is an initiator and catalyst for cortical tau pathology and subsequent tau-mediated neurodegeneration. A few of state-of-art therapeutic strategies for AD are on the basis of the amyloid hypothesis, which explains that the aggregations of Aβ and tau are not only epiphenomena, but actively contribute to the progression of AD (Ballard et al., 2011). Although Aβ-targeting therapies, such as lecanemab and donanemab, are approved by the US Food and Drug Administration (FDA), they merely moderately slow down the cognitive decline. It suggests that tau pathology and tau-mediated neurodegeneration in AD, perhaps, should be studied independently of Aβ (**Figure 1C**), which results in a therapeutic focus shift towards tau alone and the interplay between Aβ and tau (Tzioras et al., 2023).

Hence, this study introduces the amyloid hypothesis and tau, reviews the current progress in their associated studies, explores the correlated yet independent Aβ and tau pathologies, and envisages new therapeutic strategies targeting sporadic AD through a good understanding of their relationship.

## Search Strategy

The publication dates of studies cited in this review span from 1975 to 2024. All cited studies were from the PubMed database using the keywords “tau pathology in Alzheimer’s disease,” “amyloid hypothesis,” “Alzheimer’s disease,” “amyloid-beta and tau interaction,” “clinical trials for Alzheimer’s disease therapies,” “neurodegeneration,” “synaptic dysfunction in Alzheimer’s disease,” and “amyloid-beta and tau dual targeting.”

## Amyloid Hypothesis

Aβ originated from the observations in autosomal dominant AD and Down syndrome, where mutations in *APP*, *PSEN1*, and *PSEN2* unequivocally impact cerebral Aβ metabolism (Romoli et al., 2021). These mutations either elevate the production of Aβ_42_, the form closely linked to AD, or modify the Aβ_42_/Aβ_40_ ratio, which both lead to the Aβ_42_ plaque accumulation (Polanco et al., 2018). Except for a protective *APP* mutation reducing Aβ production, *APP*, *PSEN1*, and *PSEN2* exhibit nearly 100% penetrance, causing cognitive impairment to the gene carriers (Jonsson et al., 2012). Autosomal dominant AD has an estimated prevalence of less than 1%, thus most AD cases are sporadic and predominantly influenced by the apolipoprotein E (*APOE*) gene and environmental factors (Van Cauwenberghe et al., 2016). Despite the fact that the amyloid hypothesis is derived from autosomal dominant AD and Down syndrome, the hypothesis may also apply to sporadic AD (Frisoni et al., 2022).

**Figure 2:**
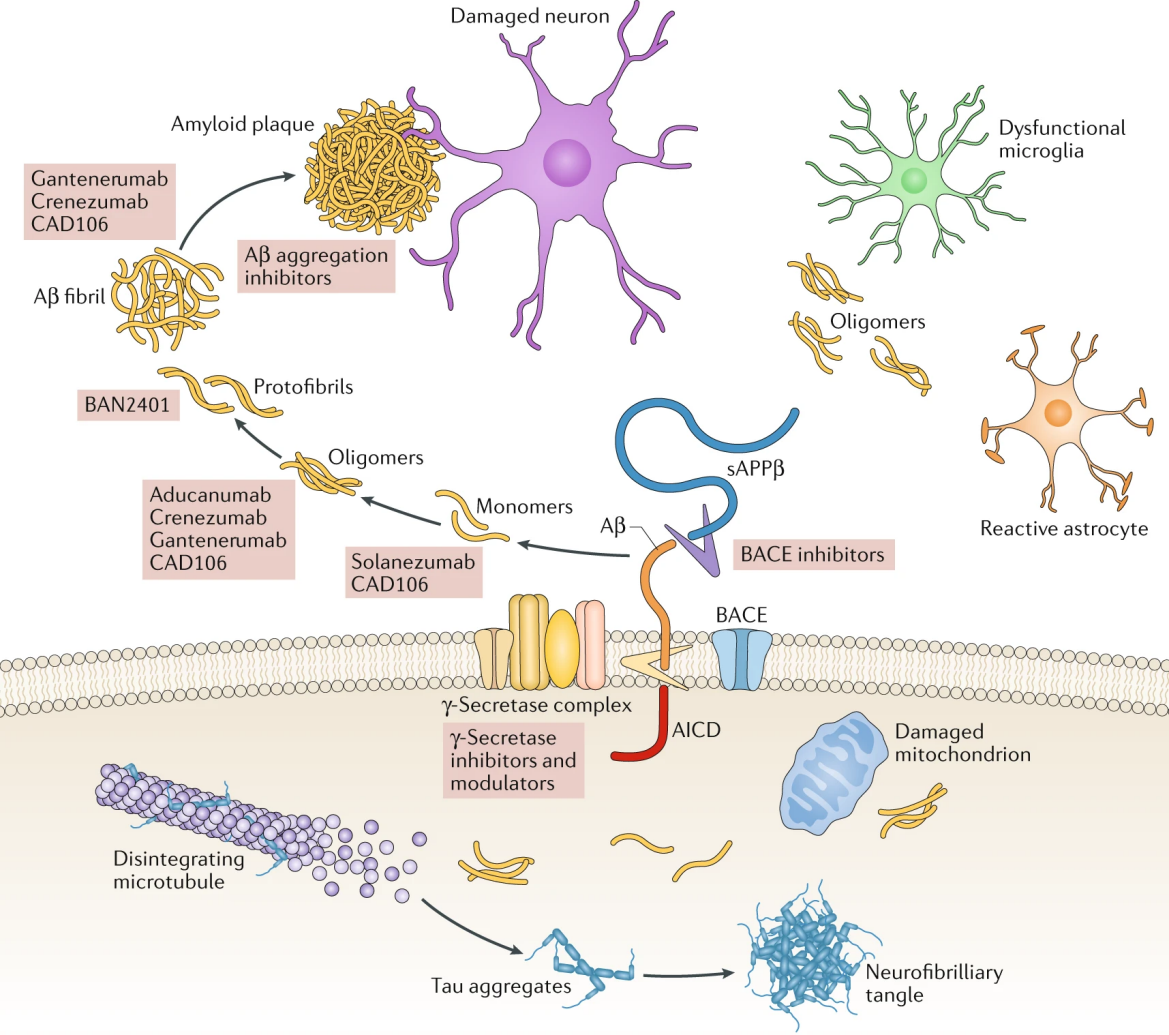
Targets and mechanisms of leading anti-Aβ drugs in Alzheimer’s disease treatment. Reprinted with permission from Panza et al. (2019). Aβ: Amyloid-β; AICD: amyloid precursor protein intracellular domain; APP: amyloid precursor protein; BACE: β-secretase; sAPPβ: soluble amyloid precursor protein β.

In the amyloid hypothesis, it is believed that malfunctions of the production, accumulation, and disposal of Aβ are the main causes of AD. In AD, β- and γ-secretases cleave APP into small fragments such as Aβ (Zhou et al., 2022). Aβ first forms small clusters called oligomers, then fibrils, β-sheets, and eventually plaques that are comprised of clumps of β-sheets and other substances (**Figure 2**). According to the hypothesis, Aβ aggregation disrupts cell-to-cell communication and activates immune cells that trigger inflammation and, ultimately, lead to the brain cell death. Meanwhile, physiologically speaking, Aβ deposition is a stepwise process beginning in the temporobasal and frontomedial areas, extending to the neocortex, primary sensory-motor areas, and the medial temporal lobe, and eventually the striatum, which was observed through the cross-sectional and longitudinal amyloid positron emission tomography scan on humans (Karran and De Strooper, 2022).

Additionally, a predominant therapeutic theory posits that the anti-Aβ monoclonal antibodies are capable of fragmenting amyloids. The swifter the fragmentation, the sooner patients would display amelioration of tau pathology and cognitive decline, albeit a lag time delay, which suggests that achieving an amyloid minimal or absent state is necessary for the brain to re-establish homeostasis (Karran and De Strooper, 2022). It is noteworthy that two types of anti-Aβ antibodies, lecanemab (Biogen and Eisai) and donanemab (Eli Lilly), have been approved by FDA for the treatment of AD among people with mild cognitive impairment. Despite the side effects, limited efficacy, and unknown long-term treatment effect and safety, the approval of these therapeutics is undoubtedly significant. It demonstrates the legitimacy of the amyloid hypothesis, offers an alternative therapeutic strategy, and paves the way for the development of more anti-Aβ drugs and/or integrative therapies. However, Aβ-targeted therapies have not been able to cure AD, and this can largely be attributed to several key factors. The foundational “amyloid hypothesis” oversimplifies the complex pathogenesis of AD. Additionally, while Aβ-targeted therapies are effective at reducing Aβ plaques, they often fail to significantly impact the more neurotoxic oligomeric form of Aβ (Timmers et al., 2019).

## Tau and Prospect of Tau-Specific Targeted Therapies

**Figure 3:**
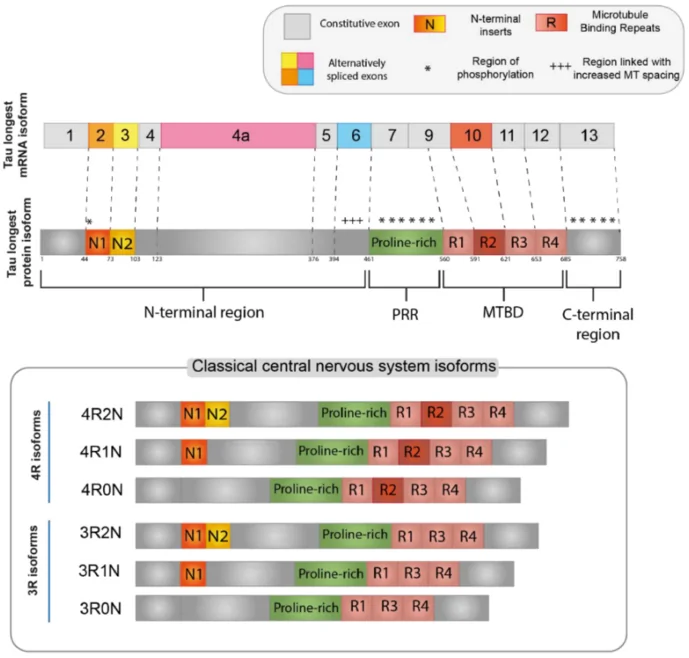
Schematic illustration of the main CNS tau protein isoforms (4R2N, 4R1N, 4R0N, 3R2N, 3R1N, and 3R0N) formed via alternative splicing. The top inset shows the longest tau isoform (4R2N) in humans that contains all the alternatively spliced exons. “4R” or “3R” differentiates the isoforms regarding the R2 segment encoded by inclusion or exclusion of exon 10, respectively. “0N”, “1N” and “2N” indicate the isoforms with N1 and N2 segments encoded in absence of both exons, exon 2 only, and both exons 2 and 3, respectively. Interestingly, in the adult human brain, the *MAPT* gene is expressed yielding equimolar 4R and 3R isoforms. Nonetheless, 0N, 1N, and 2N isoforms are expressed as 37, 54, and 9% out of total tau proteins, respectively. Reprinted with permission from Corsi et al. (2022). CNS: Central nervous system; MAPT: microtubule-associated protein tau; MTBD: microtubule-binding domain; PRR: proline-rich region.

In 1975, tau proteins were first isolated from tubulin by ion exchange chromatography, designated in short for tubulin-associated unit, and observed to be essential for microtubule assembly by Kirschner et al. (1975). In the past nearly five decades, it has been learned that tau proteins are widely present in the central nervous system (CNS), predominantly in neurons and less commonly in astrocytes and oligodendrocytes. They are comprised of isoforms generated by the alternative RNA splicing of a single gene microtubule-associated protein tau (*MAPT*) containing 16 exons located on human chromosome 17q21.31 (Neve et al., 1986; Rao et al., 2010). In the adult CNS, six major isoforms consist of different N1, N2, and R2 segments that derive from inclusion or exclusion of exons 2, 3, and 10, illustrated in **Figure 3** (Corsi et al., 2022). Additionally, more interesting isoforms of tau proteins have been discovered lately, such as Big-Tau, Exon 6, and W-Tau. Big-Tau and Exon 6 have primarily been observed in the peripheral nervous system (Wei and Andreadis, 1998; Fischer and Baas, 2020). W-Tau was reported recently by Garcia-Escudero et al. (2021) to be less prone to aggregate than the other tau isoforms. Furthermore, tau proteins have four major functional domains, as shown in **Figure 3**, namely N-terminal region, proline-rich region, microtubule-binding domain, and C-terminal region. Many functions of these domains overlap and are associated with their intramolecular interactions. Also, the six isoforms differ in size, ranging from 352 to 441 amino acids, which originates from the differences in the number of microtubule-binding repeats and amino acids near the C- and N-terminuses (Alonso et al., 2018). However, the molecular weight of the six isoforms largely varies according to different literature reference sources, although 55–62 kDa was reported by an early study (Hirokawa et al., 1988), which demands consistency. Moreover, tau proteins are intrinsically disordered proteins and lack a well-defined secondary structure (Skrabana et al., 2006). Nonetheless, a “paper-clip” tertiary structure was proposed for tau proteins (Jeganathan et al., 2006), and tau proteins can form dimers, oligomers, and larger polymers by intramolecular cysteine-cysteine links and polyanions (Huvent et al., 2014; Soeda et al., 2015). Tau proteins primarily contribute to maintaining the stability of axons of neurons by facilitating tubulin assemblies into microtubules, signal transmission between neurons, regulation of myelination, iron homeostasis, neurogenesis, and synaptic plasticity (Wang and Mandelkow, 2016; Didonna, 2020; Liang et al., 2022). They are also reported to play a role in gene expression regulation, DNA/RNA protection and metabolism, genome stability, and other protein synthesis (Corsi et al., 2022).

However, changes in the structure of MAPT by alterations of post-translational modifications (PTMs) such as hyperphosphorylation will negatively impact its functions. PTMs indicate the modifications that occur among proteins either shortly after their translations by ribosomes or after their folding and localization are complete (Alquezar et al., 2021). Once hyperphosphorylated, tau proteins can aggregate through ionic and/or hydrophobic interactions into dimers, oligomers, paired helical filaments, and insoluble NFT (Chen and Yu, 2023; Hernández et al., 2023). Nonetheless, the mechanisms for MAPT hyperphosphorylation are not fully understood, and there exists a gap on whether the toxic effect of tau results from a general increase of phosphate per protein or phosphorylation at specific sites of the protein molecules (Alonso et al., 2018). Additionally, the phosphorylation level of MAPT is considered to be dynamically regulated by tau phosphatases (for example, PP2A) and tau kinases such as glycogen-synthase kinase-3β, cyclin-dependent protein kinase 5, cAMP-dependent protein kinase, and stress-activated protein kinases (Gong and Iqbal, 2008). Thus, it is promising to reverse abnormal hyperphosphorylation of tau proteins by regulating these enzymes. Other PTMs of tau proteins include glycosylation, glycation, acetylation, ubiquitination, SUMOylation, proteolysis, methylation, protonation, oxidation, and nitration, whose roles in the tau dynamic regulations have been demonstrated but not concluded by an increasing number of studies (Alquezar et al., 2021). Additionally, similar to hyperphosphorylation, these PTMs critically regulate the assembly of unfolded tau into highly ordered β-sheet rich aggregates; however, possibly by changing the charge distribution on tau (Alquezar et al., 2021). Furthermore, PTMs can affect protein degradation by acting on the ubiquitin-proteasome system and the autophagy-lysosomes pathway, the two main protein degradation systems in cells, while protein degradation is significant for the maintenance of tau homeostasis as it prevents the accumulation of misfolded or aggregated protein species (Hipp et al., 2019). Most interestingly, current research studies suggest that there exist crosstalk, combination, and competition among different PTMs, which play key roles in tau functions, aggregation, and degradation (Alquezar et al., 2021).

Similar to Aβ, deposition of tau aggregates is a pathological hallmark of AD and other tauopathies. As the most common form of dementia, AD is considered as a secondary tauopathy considering that diagnoses of AD rely on observations of both extracellular amyloid deposition and intracellular tau aggregation, and amyloid deposition occurs earlier than the appearance of NFTs composed of both 3R and 4R tau isoforms in neurons (He et al., 2018; Götz et al., 2019; d‘Errico and Meyer-Luehmann, 2020; Chung et al., 2021). NFTs were first mentioned as a major brain lesion in AD in 1906 by Alois Alzheimer, and tau was identified as their primary component in 1985 (Götz et al., 2019; Naseri et al., 2019). Later, the relation between the NFT density and clinical symptoms of cognitive decline was revealed in AD patients (Arriagada et al., 1992; Gómez-Isla et al., 1997). Nowadays, in the context that nearly all the anti-Aβ drugs have failed to show satisfactory therapeutic efficacy, tau has received increasing attention as a potential AD therapeutic target since cognitive dysfunction has been observed to be more associated with tau than Aβ pathologies (Chen and Yu, 2023). Additionally, in comparison to insoluble tau fibrils, many studies have shown the soluble and non-fibrillar tau oligomers play a more critical role in the neurotoxicity and tau propagation from neuron to neuron via exosomes in the CNS (Jackson et al., 2022; Hyman, 2023). Nonetheless, the mechanisms that govern the spread of tau are still under investigation. Furthermore, interestingly, growing fundamental research has demonstrated a complex interplay between tau pathology and neuroinflammation (Chen and Yu, 2023). To be specific, in the early stages of AD, moderate inflammatory responses can alleviate tau pathology as activated microglia can promote the removal of tau seeds. On the contrary, in the late stages of AD, increased and sustained inflammatory responses in neurons and glia significantly advance the tau pathology. Currently, tau-targeted therapies developed for AD and other tauopathies primarily fall into gene therapy, tau PTM mediators, anti-tau immunotherapy (active and passive), tau aggregation inhibitors and microtubule stabilizers (Chen and Yu, 2023). Even though no drugs have been approved yet to treat tauopathies, a few agents targeting tau PTMs and spread have entered clinical trial phases. Tau-targeted therapies face similar challenges to amyloid-targeted ones because tau’s role in AD is complex. Normally, tau helps stabilize structures inside nerve cells that are crucial for cell health (Adams et al., 2019). However, when tau becomes abnormal and clumps into tangles, it harms cell functions (Alonso et al., 2018). Finding treatments that target only the harmful tau without affecting the healthy tau is tough. Even drugs designed to decrease tau clumping, such as tau kinase inhibitors, can accidentally affect other parts of the cell, causing unintended issues (Skrabana et al., 2006).

## Interplay between Amyloid-Beta and Tau

**Figure 4:**
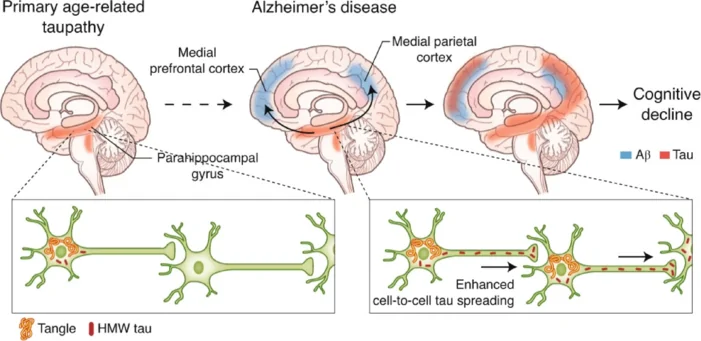
Aβ plaques expedite the propagation of tau and contribute to cognitive deterioration in AD. In cognitively normal aging, tau NFTs without cortical plaques occur in brain stem cell nuclei and the parahippocampal gyrus. In AD, cortical plaques correlate with tau spread to neocortical areas. AD patients with both plaques and tangles (right) show enhanced formation and propagation of bioactive, high molecular-weight tau compared to the patients with tau tangles alone (left). Reprinted with permission from Busche and Hyman (2020). Aβ: Amyloid-β; AD: Alzheimer’s disease; NFTs: neurofibrillary tangles.

A recent study has shown that amyloid pathology plateaued in the brain, occurring approximately two decades prior to significant cognitive impairments (Pontecorvo et al., 2019). Upon surpassing a threshold in response to amyloid pathology, there is an accelerated spread of tau pathology (Mattsson-Carlgren et al., 2020). To be specific, a recent study in cognitively normal individuals suggests that cortical Aβ increases the likelihood of tau spreading beyond the entorhinal cortex (Adams et al., 2019). A longitudinal study in elders also demonstrates that Aβ accumulation aids in the propagation of tau in the posterior cingulate cortex and the Aβ-tau interaction enhances cognitive decline (**Figure 4**; Sperling et al., 2019). Additionally, the dissemination of tau aggregates is consistently correlated with the existence of Aβ plaques, indicating that either the formation of Aβ plaques precedes that of tau tangles or that there is a synergistic interaction between Aβ plaques and tau tangles (Pontecorvo et al., 2017). Thus, anti-Aβ therapeutics may find it beneficial in inhibiting Aβ as the trigger, aiming to slow down the downstream of neurodegenerative events (De Strooper and Karran, 2016). However, numerous clinical trials aimed at decreasing Aβ have not significantly alleviated clinical symptoms, indicating that plaque removal alone may not be sufficient to slow down the AD progression (Busche and Hyman, 2020; Knopman et al., 2021; Huang et al., 2023; Ramanan et al., 2023).

A current study suggests that the synergistic effects of Aβ on tau aggregation and neuronal functions are widespread and can take place even before any visible signs of the formation of Aβ plaques and tau tangles (Busche et al., 2019). When both Aβ plaques and soluble Aβ are present, they enhance the aggregation of tau through cross-seeding (Gomes et al., 2019). Furthermore, while an early study has shown that Aβ binds tau in both *in vitro* and *in vivo* experiments, it is unclear whether this process relies on a directional interaction (DeVos et al., 2018). The synergistic association between Aβ and tau is predicted to be a biomarker for longitudinal memory decline, in which the levels of tau tangles and Aβ plaques were observed to correlate with cognitive performance (Timmers et al., 2019).

**Figure 5:**
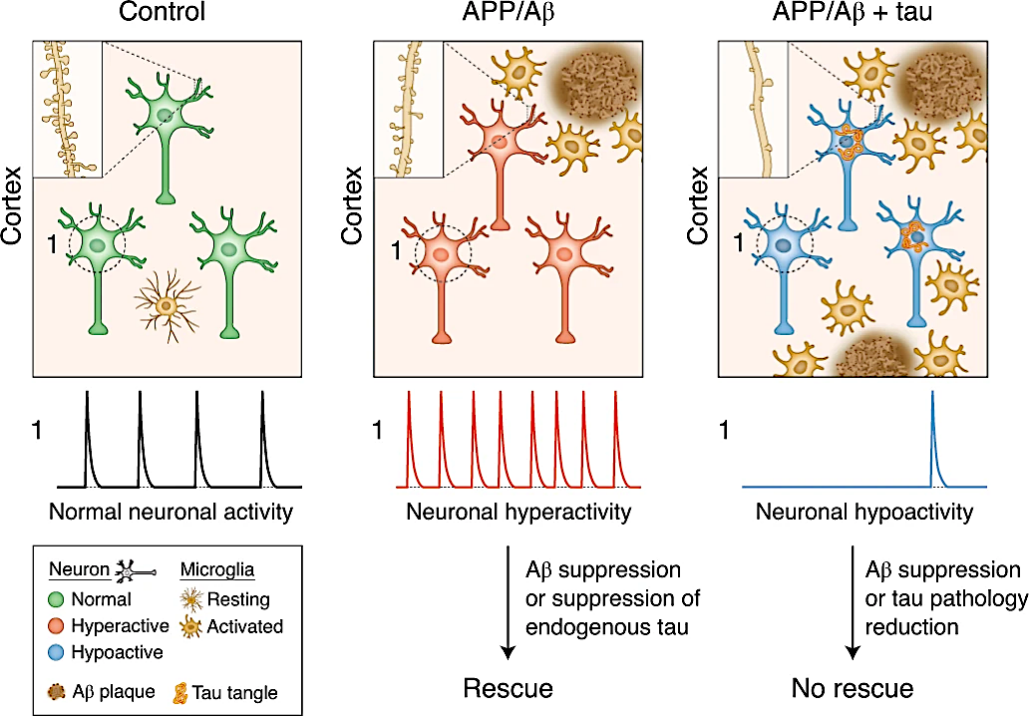
Interplay of Aβ and tau amplifies the impairment of neural circuits. Comparison between a healthy brain (left), the microenvironment around plaques (middle) and activated microglia. The coexistence of Aβ and tau in the neocortex links to reduced neuronal activity and heightened microglia activation (right). Reprinted with permission from Busche and Hyman (2020). Aβ: Amyloid-β; APP: amyloid precursor protein.

Neuronal hyperexcitability, induced by soluble Aβ, results in disrupted network oscillations, epileptiform activity, and seizures (Palop and Mucke, 2016). In multiple mechanisms, Aβ-related hyperexcitability forms are contingent on endogenous tau levels (Roberson et al., 2007). Recent findings suggest that tau can reduce the activity of brain cells even before the formation of tangles, with evidence supported by a decrease in the spontaneous action potential of brain cells in mice with tau abnormalities, revealing various ways in which tau impacts brain cell functions (Menkes-Caspi et al., 2015). The untreated chronic epilepsy seems linked to increased tau pathology in the brain (Busche and Hyman, 2020). The heightened activity in neurons leads to the release of tau to serve as seeds, inducing subsequent tau secretion and facilitating the spread of tau pathology to different regions (Wu et al., 2016). This process is also associated with disturbances in calcium levels, which can trigger harmful effects on the brain, such as changes in gene expression, local synaptic status, oxidative stress, and PTMs of tau and other proteins (Yap and Greenberg, 2018). Moreover, a study on how the co-expression of Aβ and tau impacts brain functions found that Aβ can make neurons overly active while the neuronal activity is suppressed by tau (**Figure 5**), indicating an antagonistic relationship between Aβ and tau (Busche et al., 2019). Nonetheless, some studies demonstrate a synergistic interaction between Aβ and tau in speeding up the hyperactivity caused by tau (DeVos et al., 2018).

Various factors, including changes in blood vessels, aging, lipid metabolism, and other cellular processes, show their influences on Aβ, tau, and how Aβ and tau interact in AD both independently and collectively (Sepulcre et al., 2018). These interactions lead to impaired brain functions, synaptic problems, and activation of certain enzymes. Current findings highlight multiple factors at different levels from molecular, cellular to tissue aspects of AD (Busche and Hyman, 2020), but these studies did not sufficiently consider the synergistic relationship between Aβ and tau, together with other factors. In a nutshell, a good understanding of the interplay between Aβ and tau together with other factors that affect nervous system is inevitable for understanding AD and shaping the upcoming wave of clinical trials (Polanco et al., 2018).

## Diagnostic and Therapeutic Challenges

Currently, AD diagnosis mainly stems from its heterogeneity in pathobiology (e.g., composition, location, and severity), brain integrity, varying clinical presentations, challenging timely and differential diagnoses, and lack of biomarkers or biomarker algorithms to accurately differentiate AD phenotypes (Dubois et al., 2023). The clinical phenotypes of AD include typical and atypical presentations, and atypical presentations sometimes can resemble other dementias (Paraskevas et al., 2020). Additionally, the clinical presentations of AD are modulated by co-pathologies (e.g., α-synuclein accumulation, TAR DNA-binding protein 43 pathology, vascular pathology, and non-AD tauopathies) that result in more pronounced symptoms and variations in the manifestations over time (Dubois et al., 2023). Furthermore, since neurodegenerative diseases, including AD, are likely age-related and take time to manifest, once disease onset and consequent cognitive impairment occur, all clinicians can do to help the patients is alleviating symptoms by slowing progression. Thus, a major challenge in the diagnostic and even therapeutic development of neurodegenerative diseases resides in the identification of early pathological events that will eventually lead to the death of afflicted neurons. In other words, early diagnosis with accuracy is of great significance. Another challenge in the diagnostics of AD arises along with the development and applications of biomarkers, including positron emission tomography, magnetic resonance imaging, cerebrospinal fluid concentrations of Aβ and tau proteins, and blood-based biomarkers (Dokholyan et al., 2022). While biomarkers become increasingly available and important to help distinguish among different disorders and AD phenotypes, no fluid biomarker is confidently linked to an individual clinical phenotype. These biomarkers themselves are not yet sufficient to conclusively diagnose AD or disease progression and have to be accompanied by clinical evaluation (Dubois et al., 2023). Moreover, due to the varied nature of AD, it is likely that a positive AD biomarker result does not guarantee the occurrence of AD symptoms during the lifetime of asymptomatic individuals.

Meanwhile, as of now, there are few therapies that can slow down the progression of neurodegenerative diseases. To treat mild to moderate AD, three FDA-approved drugs, galantamine (Razadyne®), rivastigmine (Exelon®), and donepezil (Aricept®), have shown promise to delay decline in memory and reduce confusion, but they cannot stop the disease from worsening over time. What is worse, patients and their families often notice no change in memory but certain side effects after taking this medicine. Additionally, these three drugs are all cholinesterase inhibitors, while drugs targeting Aβ and tau proteins are scarce, except for the fact that the FDA has given accelerated approval for two Aβ-targeting drugs, lecanemab (Lequembi®) and donanemab (Kisunla™), for people with mild symptoms. Meanwhile, memantine (Namenda®) is the only drug approved by the FDA to treat moderate to severe AD symptoms by downregulating glutamate in the brain. Ongoing attempts highlight the chronic tau-lowering strategies in AD, which can also mitigate Aβ-driven toxicity (Ng et al., 2024). To improve the drug delivery efficiency while reducing the side effects of memantine, our research group has developed a novel carbon dot-mediated memantine delivery system with significant application potentials in inhibiting tau protein aggregation while penetrating the blood–brain barrier (Zhang et al., 2022).

AD therapeutic development is severely limited by complex pathophysiology, regulations, risk-averse trial designs, delayed clinical trials due to lack of urgency, and high costs (Dokholyan et al., 2022). Due to the complex pathophysiology, the biggest obstruction in the AD therapeutic development is the unclear rooting pathological processes that cause AD dementia. In fact, it is uncertain whether Aβ and tau protein formation and aggregation are the sole or primary early pathological events, and little is known about what triggers these events and whether the death of neurons is related to these aggregates. The primary challenge in the understanding of AD etiology results from a gap between the times when various suspected pathological processes begin and when these processes manifest in symptoms. Additionally, except for the unclear root of cause, federal grant programs are highly competitive and generally in favor of the proposals of risk-averse trial designs to continue as-established research, which excludes new ideas from unconventional perspectives (Dokholyan et al., 2022).

## Concluding Remarks and Future Prospects

The modern AD therapeutic development is based on the observation that Aβ and tau exist concurrently, exhibiting a temporal connection, however, without highlighting a synergistic relationship. Compelling evidences from both experimental and clinical studies support a co-pathogenic interaction between Aβ and tau (Busche and Hyman, 2020). Despite the fact that the synergy study between Aβ and tau is still in early stages, such a synergy model can help understand the decades-long development of neurodegeneration. The development of advanced biosensors for *in situ* monitoring of cellular functional states and capable of finely tuning loss and gain of Aβ, tau, and other hallmarks of AD is imperative to precisely unravel their roles in Aβ and tau synergy. Considering the inherent complexity of most clinical systems in addressing neurological disorders such as AD, machine learning with high-resolution *in vivo* imaging technologies would help further investigate the Aβ and tau synergy.

Additionally, more tau isoforms remain to be discovered. As a robust and fast-moving area of investigation, PTMs of tau may impact the basic, pre-clinical, and clinical-translational aspects of tau biology in interesting ways for the years to come. Significantly, a few therapies targeting tau PTMs and spread are being studied in clinical trials.

Furthermore, it is particularly encouraging that two drugs targeting Aβ have recently received accelerated FDA approval for AD treatment. However, the long-term efficacy and safety of these drugs need further investigation.

Moreover, current studies indicate that aiming at single therapeutic targets may not be able to slow down or abort the pathological process of AD. Multi-target therapies (including single multi-target drugs such as p38α inhibitors, and single- or multi-target drug combination such as the combination of dasatinib and quercetin) simultaneously targeting Aβ, tau pathologies, and neuroinflammation, which are currently undergoing clinical trials. These therapeutic strategies demonstrated safety and tolerability, and the clinical trials of their therapeutic effects are underway.

In summary, both basic research and clinical trials suggest that targeting multiple pathologies and precise treatment strategies are becoming the trend of future drug development for AD, which is more likely to bring breakthroughs in the treatment of AD and other tauopathies.

## Data Availability

*Not applicable.*
